# Virulence gene profiles and molecular genetic characteristics of diarrheagenic *Escherichia coli* from a hospital in western China

**DOI:** 10.1186/s13099-018-0262-9

**Published:** 2018-08-17

**Authors:** Dan Li, Min Shen, Ying Xu, Chao Liu, Wen Wang, Jinyan Wu, Xianmei Luo, Xu Jia, Yongxin Ma

**Affiliations:** 1 Department of Medical Genetics, State Key Laboratory of Biotherapy, West China Hospital, Sichuan University, Chengdu, 610041 Sichuan China; 2 School of Medical Laboratory Science, Chengdu Medical College, Chengdu, 610500 Sichuan China; 3 Non-coding RNA and Drug Discovery Key Laboratory of Sichuan Province, Chengdu Medical College, Chengdu, 610500 Sichuan China; 4 Clinical Laboratory, The First Affiliated Hospital of Chengdu Medical College, Chengdu, 610500 Sichuan China; 5 West China School of Public Health, Sichuan University, Chengdu, 610041 Sichuan China

**Keywords:** Diarrhea, *Escherichia coli*, Virulence genes, Antimicrobial resistance, Molecular genetics

## Abstract

**Background:**

Diarrheagenic *Escherichia coli* (DEC) is one of the most important etiological agents of diarrheal diseases. In this study we investigated the prevalence, virulence gene profiles, antimicrobial resistance, and molecular genetic characteristics of DEC at a hospital in western China.

**Methods:**

A total of 110 *Escherichia coli* clinical isolates were collected from the First Affiliated Hospital of Chengdu Medical College from 2015 to 2016. Microbiological methods, PCR, antimicrobial susceptibility test, pulsed-field gel electrophoresis and multilocus sequence typing were used in this study.

**Results:**

Molecular analysis of six DEC pathotype marker genes showed that 13 of the 110 *E. coli* isolates (11.82%) were DEC including nine (8.18%) diffusely adherent *Escherichia coli* (DAEC) and four (3.64%) enteroaggregative *Escherichia coli* (EAEC). The adherence genes *fimC* and *fimH* were present in all DAEC and EAEC isolates. All nine DAEC isolates harbored the virulence genes *fyuA* and *irp2* and four (44.44%) also carried the *hlyA* and *sat* genes. The virulence genes *fyuA*, *irp2*, *cnf1*, *hlyA*, and *sat* were found in 100%, 100%, 75%, 50%, and 50% of EAEC isolates, respectively. In addition, all DEC isolates were multidrug resistant and had high frequencies of antimicrobial resistance. Molecular genetic characterization showed that the 13 DEC isolates were divided into 11 pulsed-field gel electrophoresis patterns and 10 sequence types.

**Conclusions:**

To the best of our knowledge, this study provides the first report of DEC, including DAEC and EAEC, in western China. Our analyses identified the virulence genes present in *E. coli* from a hospital indicating their role in the isolated DEC strains’ pathogenesis. At the same time, the analyses revealed, the antimicrobial resistance pattern of the DEC isolates. Thus, DAEC and EAEC among the DEC strains should be considered a significant risk to humans in western China due to their evolved pathogenicity and antimicrobial resistance pattern.

## Background

Diarrheal illnesses are the major cause of morbidity and mortality in both infants and young children and pose a severe public health problem. Diarrheal diseases are most prevalent in low- and middle-income areas in Africa, Asia, and Latin America because of poor living conditions [1, 2]. In China, infectious diarrhea continues to be one of the foremost public health issues, with up to 70,000,000 infectious diarrheal cases annually; the incidence of diarrhea is in the top three of the 38 notifiable infectious diseases [3, 4].

The bacterium *Escherichia coli* is one of the most important etiological agents of diarrheal diseases. *E. coli* strains have evolved by acquiring various functions through horizontal gene transfer, enabling them to successfully persist in hosts [2, 5, 6]. The acquisition of different groups of virulence genes has resulted in the formation of specific types of diarrheagenic *E. coli* (DEC) [5].

DEC consist of six major pathotypes: enteroaggregative *E. coli* (EAEC), enterohemorrhagic *E. coli* (EHEC; e.g., Shiga toxin-producing *E. coli*, STEC), enteropathogenic *E. coli* (EPEC), enteroinvasive *E. coli* (EIEC), enterotoxigenic *E. coli* (ETEC), and diffusely adherent *E. coli* (DAEC) [5]. EAEC is characterized by the presence of the transcriptional activator gene *aggR* and/or the serine protease precursor gene (*pic*) and/or the enteroaggregative heat stable toxin 1 (EAST-1) gene (*astA*). The presence of Shiga toxin genes (*stx1* and *stx2*) is attributed to EHEC. EPEC is characterized by the presence of the intimin gene (*eae*) and/or the bundle forming pili gene (*bfp*). The product of the *eae* gene enables attachment and effacement on intestinal epithelial cells, while *bfp* is encoded on the EPEC adherence factor (EAF) plasmid. EIEC harbors an invasion plasmid encoding several invasion genes including *ipaH*. ETEC is defined by two toxin genes, heat labile (*elt*) and/or heat-stable (*est)*. Similar to most DEC characterized, DAEC carries two F1845 fimbrial adhesion genes (*daaD* and/or *daaE*), which are highly conserved and probably involved in the virulence mechanism [2, 7, 8].

In DEC pathogenesis, adherence is generally the initial, prerequisite step in successful colonization of a specific host mucosal tissue and fimbriae play an important role in adherence [9–13]. The adherence genes examined in this study are all structural genes of different fimbriae. Type 1 fimbriae (encoded by *fimC* and *fimH*) bind to mannose-containing receptors on epithelial cells [14–16]. The aggregative adherence fimbria (AAF/I-AAF/V) family includes five types; *aggA*, *aafA*, *agg3A*, and *agg4A* encode aggregative adherence fimbria (AAF/I-AAF/IV), respectively [17–21], which mediate localized adherence, the aggregative (AA) pattern, and biofilm formation [22–24]. The long polar fimbriae (LPF) are encoded by the conservative fimbrial gene (*lpfA*) in some DEC strains [25, 26]. Additional adherent genes have been used to screen DEC including *sfa* (S fimbriae) and *pap* (P fimbriae) [27].

Following adhesion, DEC produces cytotoxic effects on the intestinal mucosa by secreting virulence factors, in order to induce mucosal inflammation [28–30]. Pathogenicity islands (PIs) are large regions of microbial genomes; in same species, they are present in pathogenic, but not in non-pathogenic strains [31]. The high pathogenicity island (HPI) appears to be widespread in *Enterobacteriaceae* [32–34]. The *irp2* and *fyuA* genes are important structural genes of HPI [35–37]. Another PI, known as the locus of enterocyte effacement (LEE), can induce attaching and effacing (AE) lesions [38]. LEE is organized in five operons (LEE1 to LEE5) [39–41] including the *escJ*, *escN*, *escV*, and *espP* structural genes [42]. In addition to LEE, various non-LEE (Nle) effectors (encoding *nleB*, *nleE*, and *ent/espL2*) [40, 43, 44] are located outside of the LEE region [45, 46]. Nle proteins contribute to increased bacterial virulence [44].

The remaining virulence factors examined in this study have been reported in previous studies. *E. coli* strains isolated in the 1980s from intestinal or extra-intestinal infections were designated as either cytotoxic necrotizing factor type 1 (CNF1) or cytotoxic necrotizing factor type 2 (CNF2) [47–49]. In 1987, an *E. coli* strain isolated from a diarrheal patient was found to possess cytolethal distending toxin (CDT) [50]. In 1990, Watanabe et al. [51] discovered the InvE protein, which is considered as an essential factor for virulence gene expression in *Shigella sonnei*. In the 1990s, α-hemolysin (HlyA) was shown to belong to a group of pore-forming leukotoxins containing RTX repeats. HlyA is a known virulence factor in *E. coli* [52–54]. In 1998, Navarro-Garcia et al. demonstrated that Pet (plasmid encoded toxin) is a cytotoxin that modifies the cytoskeleton of enterocytes, causing rounding and cell detachment in EAEC [55]. In 2001, Henderson and Nataro reported that secreted autotransporter toxin (Sat) belongs to the serine protease autotransporter subfamily of *Enterobacteriaceae* (SPATE) toxins [56]. In 2004, Paton et al. [57] revealed that some *E. coli* strains isolated from patients produced an AB_5_ toxin subtilase (SubAB).

DEC strains have been reported more and more frequently in diarrheal patients in different regions of China including Beijing [58], Shanghai [59], Henan Province [60], Wuhan [61], Kunming [62], Zhejiang Province [63] and Hongkong [64]. However, no data is available regarding DEC strains in western China and their virulence genes. Thus, in this study, we investigated the prevalence and characteristics of DEC at a hospital in western China.

## Results

### Prevalence of DEC among 110 *E. coli* strains

In order to investigate the prevalence of DEC, we categorized the clinical *E. coli* (n = 110) isolates into different DEC pathotypes based on the PCR results for virulence marker genes. Thirteen (11.82%) of the 110 *E. coli* strains were identified as DEC; nine (8.18%) and four (3.64%) of these 13 DEC strains were shown to be DAEC and EAEC, respectively. No EPEC, EHEC, ETEC, or EIEC strains were detected in this study. These results suggest the existence of a certain incidence of DEC at this hospital in western China.

### Prevalence of DAEC and EAEC among DEC

Nine of the 13 DEC isolates were DAEC, giving a positive rate of 69.23% among DEC and 8.18% among the 110 *E. coli* samples. All nine DAEC isolates were *daaD*-positive and *daaE*-negative.

The four EAEC isolates carried the *pic* gene; however, the other two EAEC virulence marker genes (*aggR* and *astA*) were not detected in any of the 110 *E. coli* strains. The positive rate of EAEC was 30.77% in DEC and 3.64% in the 110 *E. coli* samples. These results suggest that DAEC was the most common of the six major pathotypes in this study, followed by EAEC.

### Presence of adherence and virulence genes

**Fig. 1:**
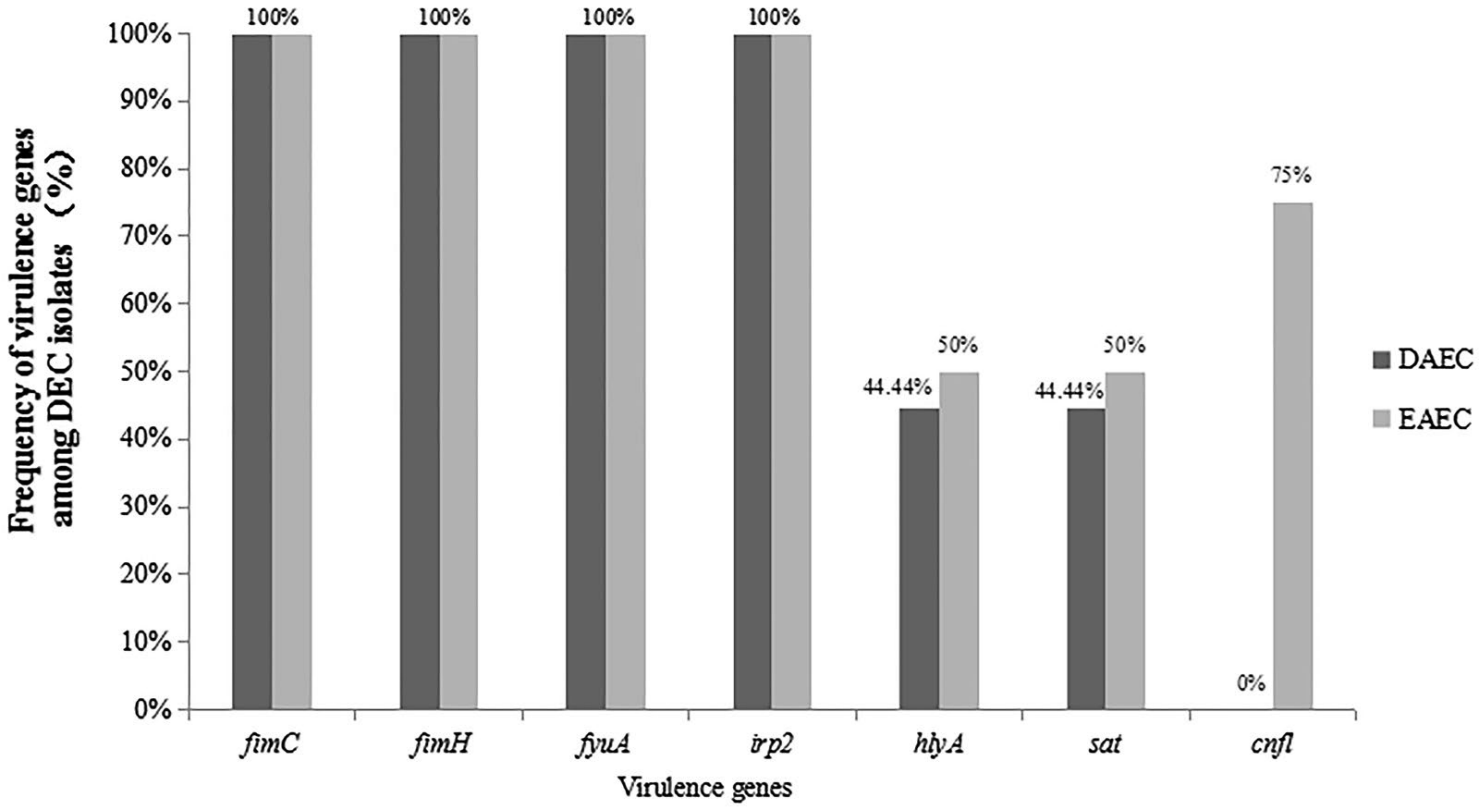
Frequency of virulence genes among DEC isolates

All DAEC and EAEC strains were tested by PCR to detect the nine adherent genes and 18 toxin-encoding genes. As shown in Table 1 and Fig. 1, all nine DAEC strains harbored the *fimC*, *fimH*, *fyuA*, and *irp2* genes (100%) and four (44.44%) also contained the *hlyA* and *sat* genes.

**Table 1:** Distribution of virulence gene incidence among DEC

DEC group	Virulence genes, *% (n)*						
	*fimC*	*fimH*	*fyuA*	*irp2*	*hlyA*	*sat*	*cnf1*
DAEC	100 (9)	100 (9)	100 (9)	100 (9)	44.44 (4)	44.44 (4)	0 (0)
EAEC	100 (4)	100 (4)	100 (4)	100 (4)	50 (2)	50 (2)	75 (3)

Concomitantly, all four EAEC strains were positive for *fimC*, *fimH*, *fyuA* and *irp2* (100%). The *cnf1* gene was identified in three (75%) EAEC strains and the *hly* and *sat* genes were both found in two (50%) of the four EAEC strains (Table 1 and Fig. 1).

All DAEC and EAEC isolates were negative for the remaining adherence and toxin-encoding genes tested (*aggA*, *aafA*, *agg3A*, *agg4A*, *lpfA*, *sfa*, *pap*, *escJ*, *escN*, *escV*, *espP*, *nleB*, *nleE*, *ent/espL2*, *cnf2*, *cdt*-*I*, *cdt*-*II*, *invE*, *pet*, and *subAB*). Therefore, our data indicate that *fimC*, *fimH*, *fyuA*, *irp2*, *hlyA*, and *sat* contribute to DAEC pathogenesis, while *fimC*, *fimH*, *fyuA*, *irp2*, *cnf1*, *hlyA*, and *sat* are involved in EAEC pathogenesis.

### Antimicrobial resistance

The antimicrobial resistance of these DEC isolates against 23 antibiotics was examined; both the DAEC and EAEC isolates exhibited high frequencies of antimicrobial resistance. All nine DAEC isolates were resistant to sulfonamide, doxycycline, and tetracycline. The resistance rates to cefotaxime, ampicillin, ticarcillin, nalidixic acid, cefoperazone, piperacillin, gentamicin, ciprofloxacin, levofloxacin, ofloxacin, tobramycin, cefoxitin, ceftazidime, minocycline, aztreonam, kanamycin, amikacin, meropenem, imipenem, and ertapenem were 88.89% (8/9), 88.89% (8/9), 88.89% (8/9), 77.78% (7/9), 66.67% (6/9), 66.67% (6/9), 55.56% (5/9), 55.56% (5/9), 44.44% (4/9), 44.44% (4/9), 33.33% (3/9), 22.22% (2/9), 22.22% (2/9), 22.22% (2/9), 22.22% (2/9), 11.11% (1/9), 0% (0/9), 0% (0/9), 0% (0/9), and 0% (0/9), respectively (Table 2).

**Table 2:** Antimicrobial resistance among DEC

Antibiotic	DAEC		EAEC	
	*%*	*n*	*%*	*n*
SSS	100	9	100	4
DOX	100	9	75	3
TET	100	9	75	3
CTX	88.89	8	25	1
AMP	88.89	8	75	3
TIC	88.89	8	75	3
NA	77.78	7	100	4
CFP	66.67	6	25	1
PIP	66.67	6	50	2
GEN	55.56	5	50	2
CIP	55.56	5	0	0
LEV	44.44	4	0	0
OFX	44.44	4	0	0
TOB	33.33	3	25	1
FOX	22.22	2	0	0
CAZ	22.22	2	0	0
MIN	22.22	2	50	2
ATM	22.22	2	0	0
KAN	11.11	1	25	1
AMK	0	0	0	0
MERO	0	0	0	0
IMP	0	0	0	0
ETP	0	0	0	0

*SSS* sulfonamide, *DOX* doxycycline, *TET* tetracycline, *CTX* cefotaxime, *AMP* ampicillin, *TIC* ticarcillin, *NA* nalidixic acid, *CFP* cefoperazone, *PIP* piperacillin, *GEN* gentamicin, *CIP* ciprofloxacin, *LEV* levofloxacin, *OFX* ofloxacin, *TOB* tobramycin, *FOX* cefoxitin, *CAZ* ceftazidime, *MIN* minocycline, *ATM* aztreonam, *KAN* kanamycin, *AMK* amikacin, *MERO* meropenem, *IMP* imipenem, *ETP* ertapenem

The resistance rates of the EAEC strains for sulfonamide, nalidixic acid, doxycycline, tetracycline, ampicillin, ticarcillin, gentamicin, minocycline, piperacillin, tobramycin, kanamycin, cefoperazone, and cefotaxime were 100% (4/4), 100% (4/4), 75% (3/4), 75% (3/4), 75% (3/4), 75% (3/4), 50% (2/4), 50% (2/4), 50% (2/4), 25% (1/4), 25% (1/4), 25% (1/4), and 25% (1/4), respectively (Table 2). All EAEC isolates were susceptible to the remaining 10 antibiotics.

Importantly, we found that all DEC isolates, including the nine DAEC and four EAEC strains, were multidrug resistant (MDR). These results suggest that clinical abuse of antibiotics is already a very serious problem in China.

### Frequency of virulence genes among antimicrobial resistant DEC isolates

Virulence gene frequencies among the antimicrobial resistant DAEC and EAEC isolates are shown in Tables 3 and 4. The frequency of the *fimC*, *fimH*, *fyuA*, and *irp2* virulence genes among resistant DEC isolates reached 100%, while the frequency of the remaining genes (*hlyA*, *sat*, and *cnf1*) among resistant isolates was mostly ≥ 50%.

**Table 3:** Frequency of virulence genes among antimicrobial resistant DAEC isolates

Antibiotic (n)	Virulence genes, *% (n)*					
	*fimC*	*fimH*	*fyuA*	*irp2*	*hlyA*	*sat*
SSS (9)	100 (9)	100 (9)	100 (9)	100 (9)	44.44 (4)	44.44 (4)
DOX (9)	100 (9)	100 (9)	100 (9)	100 (9)	44.44 (4)	44.44 (4)
TET (9)	100 (9)	100 (9)	100 (9)	100 (9)	44.44 (4)	44.44 (4)
CTX (8)	100 (8)	100 (8)	100 (8)	100 (8)	50 (4)	37.5 (3)
AMP (8)	100 (8)	100 (8)	100 (8)	100 (8)	50 (4)	37.5 (3)
TIC (8)	100 (8)	100 (8)	100 (8)	100 (8)	50 (4)	37.5 (3)
NA (7)	100 (7)	100 (7)	100 (7)	100 (7)	42.86 (3)	57.14 (4)
CFP (6)	100 (6)	100 (6)	100 (6)	100 (6)	33.33 (2)	50 (3)
PIP (6)	100 (6)	100 (6)	100 (6)	100 (6)	33.33 (2)	50 (3)
GEN (5)	100 (5)	100 (5)	100 (5)	100 (5)	40 (2)	40 (2)
CIP (5)	100 (5)	100 (5)	100 (5)	100 (5)	40 (2)	40 (2)
LEV (4)	100 (4)	100 (4)	100 (4)	100 (4)	25 (1)	50 (2)
OFX (4)	100 (4)	100 (4)	100 (4)	100 (4)	25 (1)	50 (2)
TOB (3)	100 (3)	100 (3)	100 (3)	100 (3)	0 (0)	66.67 (2)
FOX (2)	100 (2)	100 (2)	100 (2)	100 (2)	50 (1)	0 (0)
CAZ (2)	100 (2)	100 (2)	100 (2)	100 (2)	0 (0)	50 (1)
MIN (2)	100 (2)	100 (2)	100 (2)	100 (2)	50 (1)	50 (1)
ATM (2)	100 (2)	100 (2)	100 (2)	100 (2)	0 (0)	50 (1)
KAN (1)	100 (1)	100 (1)	100 (1)	100 (1)	0 (0)	100 (1)

*SSS* sulfonamide, *DOX* doxycycline, *TET* tetracycline, *CTX* cefotaxime, *AMP* ampicillin, *TIC* ticarcillin, *NA* nalidixic acid, *CFP* cefoperazone, *PIP* piperacillin, *GEN* gentamicin, *CIP* ciprofloxacin, *LEV* levofloxacin, *OFX* ofloxacin, *TOB* tobramycin, *FOX* cefoxitin, *CAZ* ceftazidime, *MIN* minocycline, *ATM* aztreonam, *KAN* kanamycin

**Table 4:** Frequency of virulence genes among antimicrobial resistant EAEC isolates

Antibiotic (n)	Virulence genes, *% (n)*						
	*fimC*	*fimH*	*fyuA*	*irp2*	*cnf1*	*hlyA*	*sat*
SSS (4)	100 (4)	100 (4)	100 (4)	100 (4)	75 (3)	50 (2)	50 (2)
NA (4)	100 (4)	100 (4)	100 (4)	100 (4)	75 (3)	50 (2)	50 (2)
DOX (3)	100 (3)	100 (3)	100 (3)	100 (3)	50 (2)	25 (1)	25 (1)
TET (3)	100 (3)	100 (3)	100 (3)	100 (9)	50 (2)	25 (1)	25 (1)
AMP (3)	100 (3)	100 (3)	100 (3)	100 (3)	50 (2)	25 (1)	25 (1)
TIC (3)	100 (3)	100 (3)	100 (3)	100 (3)	50 (2)	25 (1)	25 (1)
GEN (2)	100 (2)	100 (2)	100 (2)	100 (2)	25 (1)	0 (0)	0 (0)
MIN (2)	100 (2)	100 (2)	100 (2)	100 (2)	25 (1)	25 (1)	25 (1)
PIP (2)	100 (2)	100 (2)	100 (2)	100 (2)	25 (1)	25 (1)	25 (1)
TOB (1)	100 (1)	100 (1)	100 (1)	100 (1)	0 (0)	0 (0)	0 (0)
KAN (1)	100 (1)	100 (1)	100 (1)	100 (1)	0 (0)	0 (0)	0 (0)
CFP (1)	100 (1)	100 (1)	100 (1)	100 (1)	0 (0)	0 (0)	0 (0)
CTX (1)	100 (1)	100 (1)	100 (1)	100 (1)	0 (0)	0 (0)	0 (0)

*SSS* sulfonamide, *NA* nalidixic acid, *DOX* doxycycline, *TET* tetracycline, *AMP* ampicillin, *TIC* ticarcillin, *GEN* gentamicin, *MIN* minocycline, *PIP* piperacillin, *TOB* tobramycin, *KAN* kanamycin, *CFP* cefoperazone, *CTX* cefotaxime

### Pulsed-field gel electrophoresis

**Fig. 2:**
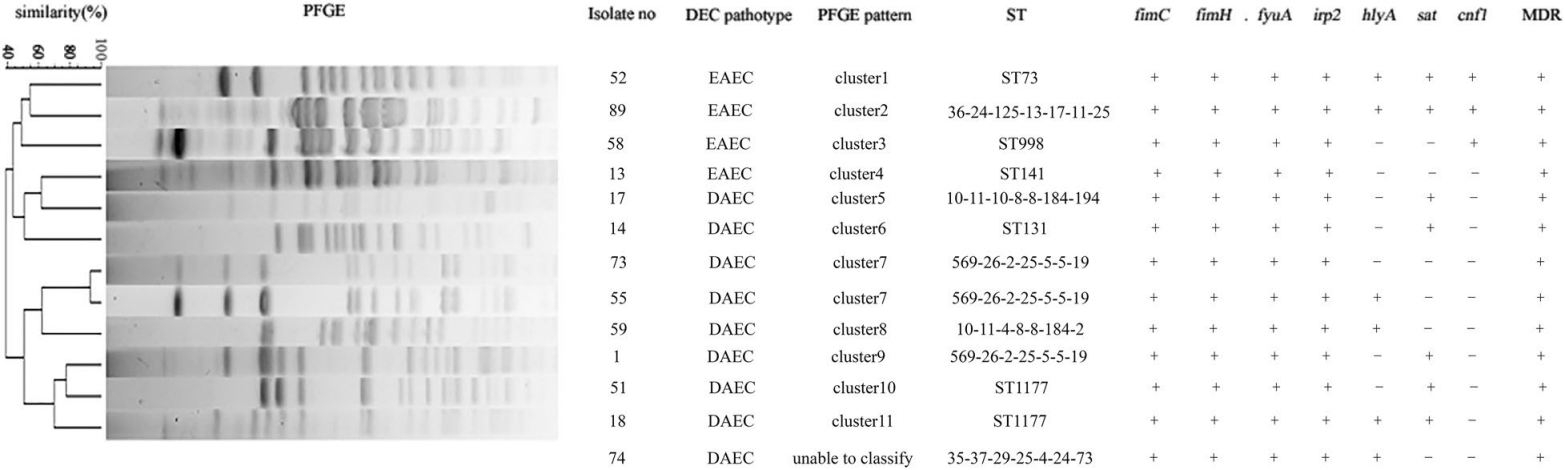
PFGE profiles of 13 DEC. The isolate number, DEC pathotype, PFGE pattern, sequence type (ST), virulence genes (*fimC*, *fimH*, *fyuA*, *irp2*, *hlyA*, *sat*, and *cnf1)* and multidrug resistance (MDR) are listed on the right. “+” indicates gene positive or MDR; “−” indicates gene negative or not MDR

The 13 DEC isolates (nine DAEC and four EAEC) were analyzed by PFGE to determine their genetic relationships. All isolates, except for no. 74, produced clear bands. The DEC PFGE results were analyzed with a Dice similarity index of 80%, according to which the 13 DEC could be divided into 11 clusters (cluster 1 to cluster 11) [65]. Isolates no. 73 and 55 belonged to one cluster, while the remaining isolates revealed another 10 distinct clusters (Fig. 2). There were no identical pulsotypes, demonstrating notable genetic diversity among the 13 DEC isolates.

### Multilocus sequence typing

The homology of the 13 DEC isolates was examined by MLST. Six of the 13 DEC isolates could be divided into five known sequence types (STs), as detailed in Fig. 2. ST1177 was the most frequent ST, represented by isolates no. 18 and 51. The remaining seven isolates could be divided into five novel STs based on their allelic profiles as detailed in Fig. 2, and are being prepared for submission. The same allelic profile (569-26-2-25-5-5-19) was detected in isolates no. 1, 55, and 73. Furthermore, the STs and PFGE patterns of the 13 DEC isolates were sporadic and heterogeneous, indicating diverse genetic backgrounds.

## Discussion

In recent years, DEC isolates have been reported in diarrheal patients in a number of studies in China; however, limited information is available regarding their prevalence in western China and virulence genes. In our study, we investigated DEC at a hospital in western China, extending our knowledge of the prevalence and characteristics of DEC in China.

The proportion of DEC among *E. coli* in our study was 11.82%, which is comparable to previous reports in Shanghai (11.6%) [66] and the Henan Province (12.05%) [60]. DEC occurrence in our study was higher than in Beijing (4.6%) [58] and the southeast coast (7.6%) of China [67]. In contrast, the detected rate of DEC was 30.2% in India [68], 39% in Brazil [69], and 30% in Peru [70], much higher than the rate in this study. These results suggest that the occurrence of DEC is comparatively low in China.

Interestingly, nine DAEC isolates were identified among the 13 DEC strains, giving a positive rate of 69.23%, indicating that DAEC was the most common major pathotype in this study. The proportion of DAEC among *E. coli* strains was 8.18% (9/110), demonstrating a certain incidence rate of DAEC at this hospital in western China. The prevalence of DAEC among *E. coli* was higher than in the neighboring Japan and in South American countries such as Peru and Colombia [70–72]. Limited information is available regarding DAEC, the sixth DEC pathotype, in China. This is the first report of the occurrence of DAEC at a hospital in western China, demonstrating that the prevalence of DAEC is comparatively high.

In the present study, 3.64% of *E. coli* isolates were EAEC, which is lower than reported in other regions in China [60, 62, 67] and much lower than reported in India, Brazil, and Peru [68–70]. However, these data show that we detected a certain level of EAEC in this study, second only to DAEC levels.

The type 1 fimbriae encoding genes *fimC* and *fimH* were identified in 100% of DAEC and EAEC isolates in our study. This adhesin is present in nearly all *E. coli* strains [34]. Lopes et al. detected *daaE*, *aggA*, *agg3A*, *sfa*, *pap*, and *fimH* in DAEC, with *fimH* the most frequently (48%) identified [73] and Lima et al. detected *agg3A*, *aafA*, *aggA*, and *agg4A* in EAEC [74]. However, we only detected the *daaD*, *fimC*, and *fimH* adherence genes, suggesting that the DAEC and EAEC strains in our study may have adhered via adhesins other than those previously described.

The HPI marker genes *fyuA* and *irp2*, first identified in *Yersinia enterocolitica*, were detected in 100% of DAEC and EAEC isolates in this study; *fyuA* and *irp2* encode the bacterial siderophore yersiniabactin. The yersiniabactin-mediated iron-uptake system is clustered in HPI and its presence is correlated with the virulence of highly pathogenic *Yersinia* [32, 75]. HPI has been shown to be widespread in various *Enterobacteriaceae* [32–34]. Therefore, it is possible that HPI could spread horizontally between *Yersinia* and DAEC/EAEC and contribute to the pathogenesis of DAEC and EAEC.

The *hlyA* gene had a positive rate of 44.44% and 50% in DAEC and EAEC, respectively. HlyA is frequently detected in EAEC and DAEC strains [23, 76]; depending on its concentration and the type of cell affected, HlyA either displays cytolytic activity or hijacks innate immune signaling pathways [54, 77, 78]. The high percent of *hlyA* in this study suggests that HlyA is involved in the mechanisms of DAEC and EAEC pathogenicity.

The *sat* gene showed a positive rate of 44.44% and 50% in DAEC and EAEC, respectively. Guignot et al. [79] have demonstrated that Sat can induce lesions on tight junctions of epithelial cells, which in turn may cause an increase in their permeability; Spano et al. [69] reported that 26.2% of DAEC and 14.5% of EAEC were positive for *sat*; Mansan-Almeida et al. [80] found that 66.7% of DAEC isolated from adult patients carried *sat*; and Lima et al. [74] identified *sat* in 38.3% of EAEC. The rate of DAEC harboring *sat* in our study is between that reported by Spano et al. and Mansan-Almeida et al., while the prevalence of *sat* in EAEC was higher than reported by Spano et al. and Lima et al. Taken together, we conclude that Sat may play a role in the pathogenesis of DAEC and EAEC.

The *cnf1* gene was found in three (75%) EAEC isolates, but not in any DAEC isolates, while *cnf2* was not detected in any DAEC and EAEC isolates. Cytotoxic necrotizing factor type 1 (CNF1) and cytotoxic necrotizing factor type 2 (CNF2) are two monomeric proteins that lead to necrosis in rabbit skin cells and multinucleation of different eukaryotic cells in culture [47, 49, 81]. Lopes et al. [73] found *cnf* in 1.8% of DAEC strains and Bouzari et al. [82] detected the *cnf1* and *cnf2* genes in 29.4% and 23.1% of DEC strains, respectively. In this study, we found *cnf1* in 23.1% (3/13) of DEC strains, but did not detect *cnf2* in any DEC strains. These results indicate that in this study the occurrence of *cnf1* and *cnf2* was lower in DEC strains, especially in DAEC.

In the current study, *pet* was not detected in any DAEC and EAEC strains. The cytotoxic mechanism of Pet arises from the degradation of α-fodrin, which is an enterocyte membrane protein [55]. Spano et al. [69] reported that 54.8% of DAEC and 55.3% of EAEC strains were positive for *pet* and Lima et al. [74] found *pet* in 10.5% of EAEC strains. These observations support our findings that few DAEC and EAEC strains in this study carry *pet*.

The antimicrobial resistance of the DAEC and EAEC strains was also examined. First-line antibiotics, such as gentamicin, cefotaxime, tetracycline, ciprofloxacin, ampicillin, and sulfonamide, showed low activity against the DAEC and EAEC strains. In particular, DAEC resistance to sulfonamide, doxycycline, and tetracycline reached 100%, while the resistance of EAEC to sulfonamide and nalidixic acid was also 100%. The resistance rates of these two pathotypes were higher than reported in developing countries including India, Brazil, and Peru [68–70]. Moreover, we found that all DAEC and EAEC isolates were MDR; only imipenem, meropenem, ertapenem, and amikacin remained effective against the nine DAEC and four EAEC isolates in this study. These results suggest that clinical abuse of antibiotics has become an increasingly serious issue in China. In addition, we found that the DEC strains not only exhibited high frequencies of antimicrobial resistance, but also showed a high frequency of carrying virulence genes (Tables 3 and 4). These properties enable DEC to successfully infect hosts and hinder effective antibiotic treatment.

Of the many genetic fingerprinting methods employed for epidemiological molecular typing, PFGE is considered to be the gold standard [83–85]. Here, using a high-resolution PFGE method, we identified a high degree of genetic diversity among the DEC isolates. Except for one isolate that we were unable to classify, we observed 11 clusters from 13 DEC isolates. None of the isolates had an identical pulsotype. These data demonstrate high genotype diversity among the DEC isolates.

MLST based on DNA sequence variations in slowly-evolving housekeeping genes has been used in epidemiological studies [86, 87]. In the present study, the 13 DEC strains could be divided into 10 STs including five novel STs. Chen et al. [86] reported that most clinical DEC isolates circulating in southeast China show a high degree of genetic diversity within a relatively small area, in agreement with our findings.

In summary, the 13 DEC isolates showed different PFGE patterns and STs, but harbored similar virulence genes (*fimC*, *fimH*, *fyuA*, *irp2*, *sat*, *hlyA*, and *cnf1*) and exhibited high antimicrobial resistance (Fig. 2). Strain phylogenetic origin changes according to ecological niche, lifestyle, and propensity to cause disease [88]. The exchange of virulence and other genes may favor such genetic relatedness. Genes associated with various pathotypes are acquired by many different DEC lineages and some lineages are more competitive than others because of the acquired virulence genes [85, 89]. In our study, the different DEC isolates exhibited diverse genotypes, but demonstrated a similar phenotype. This can be attributed to the fact that the strains harbored comparable virulence gene profiles, further indicating that virulence genes play an important role in DEC pathogenesis.

## Conclusions

This study provides the first report of DEC, including DAEC and EAEC, in western China. Our findings expand our knowledge of DEC prevalence and characteristics in China and elucidate the role of virulence genes in DEC pathogenesis. In this study, we found that the DEC strains not only exhibited high frequencies of antimicrobial resistance, but also showed a high frequency of carrying virulence genes. These properties enable DEC to successfully infect hosts and hinder effective antibiotic treatment. Furthermore, they suggest that clinical abuse of antibiotics is already a very serious issue in China. However, further investigations are needed including additional hospitals in western China and a greater number of DEC isolates.

## Methods

### Bacterial isolates

A total of 110 non-duplicated *E. coli* clinical isolates were collected from 110 different patients in various departments (gastroenterology, endocrinology, neurosurgery, and other wards) at the First Affiliated Hospital of Chengdu Medical College, Chengdu, Sichuan, China from 2015 to 2016. Isolates were identified using standard laboratory methods and the ATB New system (bioMérieux, Lyons, France). Each isolate was further verified by PCR amplification of a 369-bp internal control region from the *E. coli* marker gene *alr* [90]. All strains were stored at − 80 °C and bacteria were grown on MacConkey Agar (Oxoid, Hampshire, UK).

### Identification of DEC by PCR

All *E. coli* isolates were examined by PCR to detect the following virulence markers: *aggR*, *pic*, and *astA* for EAEC; *stx1* and *stx2* for EHEC; *eae* and *bfp* for EPEC; *ipaH* (invasion plasmid antigen H) for EIEC; *est* and *elt* (enterotoxins) for ETEC; and *daaD* and *daaE* for DAEC. The primers used to amplify these genes are listed in Table 5.

**Table 5:** Gene primers used in this study

Gene	Primer sequence (5′-3′)	PCR product (bp)	References
*alr*	F: CTGGAAGAGGCTAGCCTGGACGAG R: AAAATCGCCACCGGTGGAGCGATC	369	[90]
*pic*	F: GGGTATTGTCCGTTCCGAT R: ACAACGATACCGTCTCCCG	1176	[93]
*astA*	F: CCATCAACACAGTATATCCGA R: GGTCGCGAGTGACGGCTTTGT	111	[73]
*aggR*	F: ACGCAGAGTTGCCTGATAAAG R: AATACAGAATCGTCAGCATCAGC	400	[94]
*stx1*	F: CGATGTTACGGTTTGTTACTGTGACAGC R: AATGCCACGCTTCCCAGAATTG	244	[94]
*stx2*	F: GTTTTGACCATCTTCGTCTGATTATTGAG R: AGCGTAAGGCTTCTGCTGTGAC	324	[94]
*eae*	F: TGAGCGGCTGGCATGAGTCATAC R: TCGATCCCCATCGTCACCAGAGG	241	[95]
*bfp*	F: GACACCTCATTGCTGAAGTCG R: CCAGAACACCTCCGTTATGC	324	[94]
*ipaH*	F: GTTCCTTGACCGCCTTTCCGATACCGTC R: AAAATCGCCACCGGTGGAGCGATC	619	[7]
*est*	F: ATTTTTCTTTCTGTATTGTCTT R: CACCCGGTACAGGCAGGATT	190	[96]
*elt*	F: GGCGACAGATTATACCGTGC R: CGGTCTCTATATTCCCTGTT	450	[96]
*daaD*	F: TGAACGGGAGTATAAGGAAGATG R: GTCCGCCATCACATCAAAA	444	[97]
*daaE*	F: GAACGTTGGTTAATGTGGGGTAA R: TATTCACCGGTCGGTTATCAGT	542	[8]
*fimC*	F: GGGTAGAAAATGCCGATGGTG R: CGTCATTTTGGGGGTAAGTG	477	[98]
*fimH*	F: CGAGTTATTACCCTGTTTGCTG R: ACGCCAATAATCGATTGCAC	878	[73]
*aggA*	F: GCTAACGCTGCGTTAGAAAGACC R: GGAGTATCATTCTATATTCGCC	421	[73]
*aafA*	F: ATGTATTTTTAGAGGTTGAC R: TATTATATTGTCACAAGCTC	518	[20]
*agg3A*	F: GTATCATTGCGAGTCTGGTATTCAG R: GGGCTGTTATAGAGTAACTTCCAG	462	[73]
*agg4A*	F: TGAGTTGTGGGGCTAYCTGGACACC R: ATAAGCCGCCAAATAAGC	169	[74]
*lpfA*	F: AGGCGGTGCATTCACTCTGGCATCT R: CCGCGTCGATAGCGGTATAGGCAGA	446	[99]
*sfa*	F: CTCCGGAGAACTGGGTGCATCTTAC R: CGGAGGAGTAATTACAAACCTGGCA	408	[73]
*pap*	F: GACGGCTGTACTGCAGGGTGTGGCG R: ATATCCTTTCTGCAGGGATGCAATA	328	[73]
*irp2*	F: AAGGATTCGCTGTTACCGGAC R: TCGTCGGGCAGCGTTTCTTCT	264	[100]
*fyuA*	F: TGATTAACCCCGCGACGGGAA R: CGCAGTAGGCACGATGTTGTA	785	[34]
*escJ*	F: CACTAAGCTCGATATATAGAACCC R: GTCAATGTTGATGTCGTATCTAAG	824	[80]
*escN*	F: CGCCTTTTACAAGATAGAAC R: CATCAAGAATAGAGCGGAC	854	[101]
*escV*	F: GATGACATCATGAATAAACTC R: GCCTTCATATCTGGTAGAC	2128	[80]
*espP*	F: AAACAGCAGGCACTTGAACG R: GGAGTCGTCAGTCAGTAGAT	1830	[93]
*nleB*	F: GGAAGTTTGTTTACAGAGACG R: AAAATGCCGCTTGATACC	297	[43]
*nleE*	F: GTATAACCAGAGGAGTAGC R: GATCTTACAACAAATGTCC	260	[43]
*ent/espL2*	F: GAATAACAATCACTCCTCACC R: TTACAGTGCCCGATTACG	233	[43]
*cnf1*	F: GGCGACAAATGCAGTATTGCTTGG F: GACGTTGGTTGCGGTAATTTTGGG	552	[93]
*cnf2*	F: GTGAGGCTCAACGAGATTATGCACTG R: CCACGCTTCTTCTTCAGTTGTTCCTC	839	[93]
*cdt*-*I*	F: CAATAGTCGCCCACAGGA R: ATAATCAAGAACACCACCAC	412	[102]
*cdt*-*II*	F: GAAAGTAAATGGAATATAAATGTCCG R: TTTGTGTTGCCGCCGCTGGTGAAA	556	[102]
*invE*	F:CGATCAAGAATCCCTAACAGAAGAATCAC R: CGATAGATGGCGAGAAATTATATCCCG	766	[94]
*hlyA*	F: GCATCATCAAGCGTACGTTCC R: AATGAGCCAAGCTGGTTAAGCT	533	[100]
*pet*	F: TTTCCAGCACTTCCTGTTCC R: ATTTCCAACGTCTACGCCAT	297	[103]
*sat*	F: GCAGCAAATATTGATATATCA R: GTTGTTGACCTCAGCAAGGAA	2913	[80]
*subAB*	F: TATGGCTTCCCTCATTGCC R: TATAGCTGTTGCTTCTGACG	556	[104]

### Detection of adherence and virulence genes

All DEC isolates were subjected to PCR to detect nine adherence genes (*fimC*, *fimH*, *aggA*, *aafA*, *agg3A*, *agg4A*, *lpfA*, *sfa*, and *pap*) and 18 virulence genes (*irp2*, *fyuA*, *escJ*, *escN*, *escV*, *espP*, *nleB*, *nleE*, *ent/espL2*, *cnf1*, *cnf2*, *cdt*-*I*, *cdt*-*II*, *invE*, *hlyA*, *pet*, *sat*, and *subAB*). The primers used to amplify these genes are listed in Table 5.

### Antimicrobial susceptibility testing

The minimal inhibitory concentration (MIC) of 23 antimicrobial agents for DEC were determined by the agar dilution methods according to the 2017 Clinical and Laboratory Standards Institute guidelines [91]. We tested the following 23 antimicrobial agents: sulfonamide, doxycycline, tetracycline, cefotaxime, ampicillin, ticarcillin, nalidixic acid, cefoperazone, piperacillin, gentamicin, ciprofloxacin, levofloxacin, ofloxacin, tobramycin, cefoxitin, ceftazidime, minocycline, aztreonam, kanamycin, amikacin, meropenem, imipenem, and ertapenem. The results were used to classify isolates as resistant or susceptible to a particular antibiotic using standard reference values [91].

### Pulsed-field gel electrophoresis (PFGE)

Genomic DNA from the DEC isolates were digested with *Xba*I and separated by PFGE according to the protocol of the Centers for Disease Control and Prevention (http://www.cdc.gov/pulsenet/pathogens/index.html). Gel images were captured with the Gel Doc XR+ system (Bio-Rad, Hercules, CA, USA). An unweighted pair-group method with arithmetic mean (UPGMA) dendrogram was constructed using BioNumerics software (Applied Maths, Sint-Martens-Latem, Belgium).

### Multilocus sequence typing

All DEC isolates were analyzed by multilocus sequence typing (MLST) according to the MLST website (http://mlst.warwick.ac.uk). Briefly, the internal fragments of seven housekeeping genes (*adk*, *fumC*, *gyrB*, *icd*, *mdh*, *purA*, and *recA*) were amplified by PCR [92] and their sequences were compared with existing sequences in the MLST database for the assignment of allelic numbers. Sequence types (STs) were assigned according to the allelic profiles.

## Abbreviations

DEC: diarrheagenic *E. coli*
EAEC: enteroaggregative *E. coli*
EHEC: enterohemorrhagic *E. coli*
EPEC: enteropathogenic *E. coli*
EIEC: enteroinvasive *E. coli*
ETEC: enterotoxigenic *E. coli*
DAEC: diffusely adherent *E. coli*
SSS: sulfonamide
DOX: doxycycline
TET: tetracycline
CTX: cefotaxime
AMP: ampicillin
TIC: ticarcillin
NA: nalidixic acid
CFP: cefoperazone
PIP: piperacillin
GEN: gentamicin
CIP: ciprofloxacin
LEV: levofloxacin
OFX: ofloxacin
TOB: tobramycin
FOX: cefoxitin
CAZ: ceftazidime
MIN: minocycline
ATM: aztreonam
KAN: kanamycin
AMK: amikacin
MERO: meropenem
IMP: imipenem
ETP: ertapenem
MDR: multidrug resistant
PFGE: pulsed-field gel electrophoresis
MLST: multilocus sequence typing
